# Rapid Detection and Differentiation of Swine-Origin Influenza A Virus (H1N1/2009) from Other Seasonal Influenza A Viruses

**DOI:** 10.3390/v4113012

**Published:** 2012-11-09

**Authors:** Jiangqin Zhao, Xue Wang, Viswanath Ragupathy, Panhe Zhang, Wei Tang, Zhiping Ye, Maryna Eichelberger, Indira Hewlett

**Affiliations:** 1 Laboratory of Molecular Virology, Center for Biologics Evaluation and Research, Food and Drug Administration, Bethesda, MD 20892, USA; Email: xue.wang@fda.hhs.gov (X.W.); viswanath.ragupathy@fda.hhs.gov (V.R.); panhe.zhang@fda.hhs.gov (P.Z.); annietang_93@yahoo.com (W.T.); 2 Division of Viral Products, Office of Vaccines Research and Review, CBER, Food and Drug Administration, Bethesda, MD 20892, USA; Email: zhiping.ye@fda.hhs.gov (Z.Y.); maryna.eichelberger@fda.hhs.gov (M.E.)

**Keywords:** nanoparticle, H5N1, swine influenza A virus, nanomicroarray

## Abstract

We previously developed a rapid and simple gold nanoparticle(NP)-based genomic microarray assay for identification of the avian H5N1 virus and its discrimination from other influenza A virus strains (H1N1, H3N2). In this study, we expanded the platform to detect the 2009 swine-origin influenza A virus (H1N1/2009). Multiple specific capture and intermediate oligonucleotides were designed for the matrix (M), hemagglutinin (HA), and neuraminidase (NA) genes of the H1N1/2009 virus. The H1N1/2009 microarrays were printed in the same format as those of the seasonal influenza H1N1 and H3N2 for the HA, NA, and M genes. Viral RNA was tested using capture-target-intermediate oligonucleotide hybridization and gold NP-mediated silver staining. The signal from the 4 capture-target-intermediates of the HA and NA genes was specific for H1N1/2009 virus and showed no cross hybridization with viral RNA from other influenza strains H1N1, H3N2, and H5N1. All of the 3 M gene captures showed strong affinity with H1N1/2009 viral RNA, with 2 out of the 3 M gene captures showing cross hybridization with the H1N1, H3N2, and H5N1 samples tested. The current assay was able to detect H1N1/2009 and distinguish it from other influenza A viruses. This new method may be useful for simultaneous detection and subtyping of influenza A viruses and can be rapidly modified to detect other emerging influenza strains in public health settings.

## 1. Introduction

Influenza viruses are classified as A, B, and C based on antigenic differences in their nucleoprotein (NP) and matrix (M) protein. Influenza A viruses are classified into 16 HA subtypes (H1–H16) and 9 NA subtypes (N1–N9) based on the antigenicity of the two surface glycoproteins: hemagglutinin (HA) and neuraminidase (NA). All subtypes of influenza A virus are found in aquatic birds, while only a subset cause disease in pigs, horses, dogs, sea mammals, and humans [1,2,3]. HA subtypes (H1, H2, and H3) and two NA subtypes (N1 and N2) have caused human pandemics [4,5]. Influenza viruses of H5 and H7 subtypes are highly pathogenic to domestic fowl due to the presence of a multibasic tryptic cleavage site in the HA [6,7]. Although it is not easily transmissible between people, avian H5N1 viruses have infected humans with significant rate of mortality since 1997 [8]. In addition to direct infection of man by avian viruses, novel genotypic reassortants in an intermediary host may result in the emergence of a novel pandemic strain. In mid-March 2009, a novel swine-origin influenza virus entered the human population and spread rapidly around the world. WHO received global data for large numbers of cases and deaths due to this 2009 swine-origin influenza virus (H1N1/2009) early on in the pandemic [9], and the virus has continued to circulate in humans, replacing the previously circulating seasonal H1N1 viruses. Continued reassortment of H1N1/2009 with swine influenza viruses may produce variants with transmissibility and altered virulence for humans [10,11]. To identify emerging viruses with pandemic potential, molecular virological monitoring needs to be implemented as a means to assess the extent to which novel strains are circulating in a population. 

Rapid subtyping of influenza A viruses, such as the pandemic H1N1/2009 and the avian influenza H5N1 viruses, is crucial to initiate adequate protective measures to prevent the spread of highly pathogenic influenza A viruses. At the present time, the most commonly used diagnostic and subtyping methods are time consuming, expensive, and inaccurate. By using a nanoparticle(NP)-based PCR-free genomic microarray assay (nanomicroarray), we previously developed a H5N1 microarray to rapidly and specifically detect H5N1 viral RNA [12]. In this study we describe the development of the nanomicroarray assay for rapid detection and subtyping of the H1N1/2009 influenza virus that discriminates between this and other seasonal influenza A viruses.

## 2. Results and Discussion

**Figure 1:**
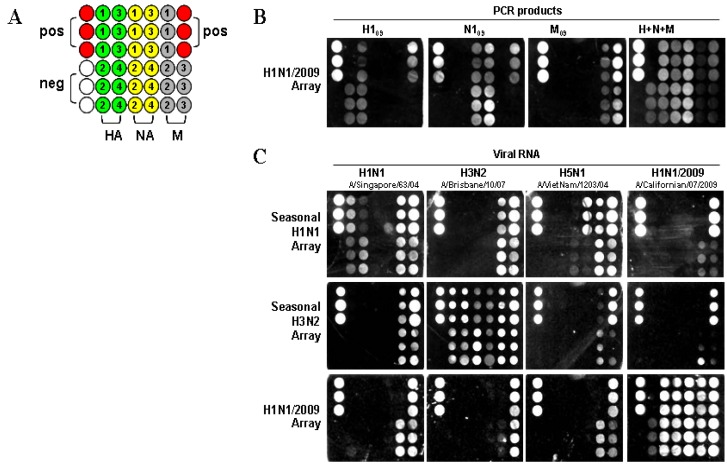
Image from a Verigene ID^TM^ detection system. (**A**) Nanomicroarray layout with positive control capture (closed red circles), negative control (closed blank circles), M, HA, and NA gene captures (filled as variable color of closed circles, spotted in triplicate) are indicated. The numbers indicate the different capture sequences from a gene shown in Table 1. Three types of arrays for H1N1, H3N2, and H1N1/2009 are printed in the same format as indicated. (**B**) Silver staining image for individual or multiple H1N1/2009 PCR products are shown and labeled. (**C**) Silver staining image from 0.5 μg of viral RNA for H1N1, H3N2, H5N1, and H1N1/2009 are shown.

To verify the ability to detect H1N1/2009 viral RNA, the nanomicroarray was initially designed and evaluated using PCR amplicons for the H1N1/2009 H1, N1, and M genes (H1_09_, N1_09_, M_09_). The assay was performed by direct hybridization of PCR products with the capture and the intermediate oligonucleotides to form sandwich complexes, followed by silver staining in a format shown in Figure 1A, with each H1N1/2009 capture (swc) oligonucleotide, listed in Table 1, spotted in triplicate. As shown in Figure 1B, PCR-amplified H1_09_, N1_09_, and M_09_ genes resulted in signals that corresponded with the capture oligonucleotide, showing specificity of the assay, and providing a unique fingerprint for each gene segment. The inability of capture swcH1 to detect the H1_09_ PCR amplicon is likely reflective of incorrect oligonucleotide synthesis. Analytical specificity was demonstrated by mixing the three DNA products simultaneously during hybridization. The signal patterns observed for the H1_09_, N1_09_, and M_09_ genes when used either in separate assays or together, were the same, although in the mixture, the M_09_ gene had low intensity. When the PCR products of the HA and NA genes for the seasonal H1N1, H3N2, and H5N1 subtypes were tested in a H1N1/2009 array, no signal was detected (data not shown). This study indicates that the H1N1/2009 array is highly specific for the detection of HA and NA genes from only the swine-origin influenza A virus. 

To determine the ability to differentiate seasonal H1N1 and H3N2 from the H1N1/2009 subtype, three types of nanomicroarray were generated. RNA samples extracted from seasonal H1N1, H3N2, avian H5N1, and H1N1/2009 viruses listed in the Experimental Section were tested in those arrays (Figure 1C). In the seasonal H1N1 array, only the HA gene from A/Singapore/63/04 (H1N1) was detected but not from the H1N1/2009 strain, A/Californian/07/2009. The NA gene from A/VietNam/1203/04 (H5N1) and A/Singapore/63/04 (H1N1) were detected by this array, but there was no cross-reactivity with the H1N1/2009 NA gene. In the seasonal H3N2 array, all three genes (H3, N2, and M) were detected in the A/Brisbane/10/07 (H3N2) sample. 

In the H1N1/2009 array, viral RNA extracted from three H1N1/2009 strains (A/California/04/2009, A/California/07/2009 and A/California/08/2009) were tested and a similar signal pattern was observed. As shown in Figure 1C, all three genes (H1_09_, N1_09_, and M_09_) from the A/California/07/2009 (H1N1) sample displayed strong signals. The three H1_09_ capture oligonucleotides (swcH2, swcH3 and swcH4) and four N1_09_ capture oligonucleotides (swcN1, swcN2, swcN3 and swcN4) could efficiently detect H1N1/2009 strain. There was only one capture swcH1 that did not produce any signal as described above in PCR amplicons suggesting that errors may have occurred in this capture oligonucleotide, a new synthesized capture swcH1 needs further evaluation. The array format described here was specifically designed to evaluate individual design of capture oligonucleotides, synthesis and printing for detection of H1N1/2009. The array has since been printed multiple times and was shown to be highly reproducible between independent arrays. This assay has a feature for detection of a specific target gene (*i.e.*, H1_09_) through multiple capture oligonucleotides and does not rely on any single capture such as swcH1. The functional capture oligonucleotides evaluated in this study were used in the development of a multiplex nanomicroarray for detection and differentiation of influenza A from B and subtyping influenza A viruses. 

**Table 1:** Oligonucleotide sequences for H1N1/2009 capture (swc), intermediate (swi) and PCR.

Oligo ID	Gene	Purpose	Sequences (5' to 3')
swcM1	M	capture	CTGACTAAGGGAATTTTAGGATTTGTGTTCACGCTCACCG
swcM2	M	capture	TGATTCACAGCATCGGTCTCACAGACAGATGGCTACTACC
swcM3	M	capture	AAGGCTATGGAACAGATGGCTGGATCGAGTGAACAGGCAG
swcH1	HA	capture	GAAGGCAATACTAGTAGTTCTGCTATATACATTTGCAACC
swcH2	HA	capture	CCTGGGAAATCCAGAGTGTGAATCACTCTCCACAGCAAGC
swcH3	HA	capture	GTTTTTGTGGGGTCATCAAGATACAGCAAGAAGTTCAAGC
swcH4	HA	capture	GAAGCAAAATTAAACAGAGAAGAAATAGATGGGGTAAAGC
swcN1	NA	capture	TTCGGTCTGTATGACAATTGGAATGGCTAACTTAATATTA
swcN2	NA	capture	GTGGTTTCCGTGAAATTAGCGGGCAATTCCTCTCTCTGCC
swcN3	NA	capture	CAATGGAACCATTAAAGACAGGAGCCCATATCGAACCCTA
swcN4	NA	capture	ACGCCCTAATGATAAGACAGGCAGTTGTGGTCCAGTATCG
swiM1	M	intermediate	AAAGCCGAGATCGCGCAGAGACTGGAAAGTGTCTTTGCAG *
swiM2	M	intermediate	CCACTAATCAGGCATGAAAACAGAATGGTGCTGGCTAGCA *
swiM3	M	intermediate	GCCTGAGTCCATGAGGGAAGAATATCAACAGGAACAGCAG *
swiM4	M	intermediate	AATGGGAGTGCAGATGCAGCGATTCAAGTGATCCTCTCGT *
swiH1	HA	intermediate	AAGACAAGCATAACGGGAAACTATGCAAACTAAGAGGGGT *
swiH2	HA	intermediate	GTGGAAACACCTAGTTCAGACAATGGAACGTGTTACCCAG *
swiH3	HA	intermediate	AAACGGCTGCTTTGAATTTTACCACAAATGCGATAACACG *
swiN1	NA	intermediate	CTTGGGAATCAAAATCAGATTGAAACATGCAATCAAAGCG *
swiN2	NA	intermediate	GTAAAGACAACAGTGTAAGAATCGGTTCCAAGGGGGATGT*
swiN3	NA	intermediate	CAGTCGCTTGGTCAGCAAGTGCTTGTCATGATGGCATCAA *
swiN4	NA	intermediate	TACGGCAATGGTGTTTGGATAGGGAGAACTAAAAGCATTA *
ABI04mF	M	PCR	TAACCGAGGTCGAAACGTA
ABI04mR	M	PCR	TTACTCTAGCTCTATGTTGACAAA
ABI05hF	HA	PCR	ATGAAGGCAATACTAGTAGTTCTGCT
ABI05hR	HA	PCR	TTCTACACTGTAGAGACCCATTAG
ABI06nF	NA	PCR	ATGAATCCAAACCAAAAGATAATAA
ABI06nR	NA	PCR	TTACTTGTCAATGGTAAATGGCAACT

* 25-mer poly (A) tail added at 3' end of each intermediate oligonucleotide.

We observed that the matrix genes of each virus subtype tested were detected in all nanomicroarrays (seasonal H1N1, seasonal H3N2 and H1N1/2009) with varying signal profiles, and that the H1N1/2009 viral RNA could hybridize to the M02 and M03 capture oligonucleotides in the seasonal H1N1 and H3N2 arrays to produce a visible signal, indicating that cross hybridization occurred as would be expected [12]. We did not test H1N1/2009 samples from other geographical regions as it was difficult to obtain specimens during the study. However since the multiple capture oligonucleotides were designed from highly conserved sequences among the H1_09 _and N1_09_ genes, we predict that the current H1N1/2009 array is highly likely to detect pandemic influenza H1N1/2009 not only from California but also from other geographic regions. In this proof of concept experiment, we demonstrate that the H1N1/2009 array is highly specific for the detection of swine-origin influenza A virus and can be used to differentiate between H1N1/2009 and H1N1, H3N2 and H5N1 viruses. 

Considering that influenza viruses can evolve rapidly and are capable of adapting and generating diversity through cross-species transmission and genetic mutation, their ability to adapt raises the risk of the emergence of novel influenza viruses to which humans are susceptible [10,13,14]. The identification of subtypes that are endemic in zoonotic sources or novel influenza strains that have pandemic potential is of high concern. The development of new virus detection systems is always important in reducing the risk of transmitted infectious diseases. However, it is extremely challenging to design assays that cover varying virus populations and newly emerging viral subtypes for rapid and accurate detection. We developed a PCR-free nanomicroarray detection platform using a capture/intermediate oligonucleotide strategy, allowing the coverage of a wide range of influenza A virus subtypes including H1N1, H3N2, and H5N1. The multiple capture and intermediate oligonucleotides are designed in conserved regions of each of the three genes spanning the whole genome in each subtype to ensure the target genes can be captured and detected, despite variations among the different strains. This type of 40 bp oligonucleotide is able to detect minor viral variants with high specificity. We employed multiplexed intermediate oligonucleotides for many of the influenza A viruses to capture and detect the specific influenza virus subtypes, mitigating the risk of unidentified mutations. Although the current nanomicroarray method is less sensitive than the Taqman and PCR assay, this assay appears to possess the potential to be used as a method for the rapid detection, subtyping, and screening of newly emerging influenza viruses in one simple assay. This assay has many advantages since it is not limited by the need for bioenzymatic-based gene amplification, sequencing, culturing, or HA/NA subtyping of emerging strains.

The genetic differences between the HA and NA gene segments of H1N1/2009 and other H1N1 strains [10,15] was significant enough to allow us to generate the multiple specific capture and intermediate oligonucleotides so that the H1N1/2009 virus can easily and rapidly be distinguished from other H1N1, H3N2, and H5N1 viruses. Although the nanomicroarray images can be scanned for further statistical analysis, conclusions on virus identity can be drawn from results seen as a specific intensity pattern by the naked eye. Our results therefore suggest this nanoparticle-based genomic microarray may provide a practical tool for virus identification, and may be particularly useful during emerging influenza pandemics. 

## 3. Experimental Section

### 3.1. Designing Oligonucleotides

Multiple capture and intermediate oligonucleotides complementary to the conserved regions of the M, H1, H3, H5, N1, and N2 genes for the H1N1, H3N2, and H5N1 influenza A viruses were designed as previously described [12]. Since the HA (H1_09_) and NA (N1_09_) genes of H1N1/2009 are 27.2% and 18.2% different in amino acid sequence, respectively, to the 2008 H1N1 strain [15], we selected 133 and 139 known sequences of H1_09_ and N1_09_ gene, respectively, including sequences of viruses from different geographical regions (*i.e.*, Northern American, Europe, and Asia) and applied 500 hit count analysis for each gene using Blast Search program in Vector NTI Advance^TM^ 11 (Invitrogen, Foster City, CA, USA). The sequence alignment using the MEGA 4 program indicated that the oligonucleotides sequences we designed were highly conserved among the H1_09_ and N1_09 _genes. The nucleotide sequences of H1 and H1_09_ or N1 and N1_09_ were further aligned to identify significant divergent regions. The specific capture and intermediate oligonucleotides unique in H1N1/2009 were designed to diagnose and distinguish the H1N1/2009 from seasonal H1N1 influenza A viruses. A total of 11 specific capture and positive control oligonucleotides were synthesized and printed on the microarray chip. Three or four intermediate oligonucleotides that bind to sequences adjacent to the capture sequence for each target gene were designed and synthesized. To validate the H1N1/2009 nanomicroarry assay, the PCR primers for the H1_09_, N1_09_, and M_09_ genes of H1N1/2009 were also generated to amplify full-length DNA fragments. The oligonucleotides sequences are listed in Table 1.

### 3.2. Samples, Viral RNA Extraction and RT-PCR

Influenza virus isolates A/Singapore/63/04 (H1N1), A/Hong Kong/8/68(H3N2), A/Brisbane/10/2007(H3N2), A/Vietnam/1203/04 (H5N1), were prepared at CBER, FDA using appropriate safety protocols. Swine-origin influenza A viruses A/California/04/2009 (H1N1), A/California/07/2009 (H1N1), A/California/08/2009 (H1N1), and the plasmids pHW-PR8 M (H1N1), pCR-II Topo HA (H5N1) and pCR-II Topo NA (H5N1) for HA, NA and M genes were provided by CDC (CDC, Atlanta, GA, USA). Some influenza virus strains were purchased from ZeptoMetrix (ZeptoMetrix corp., Buffalo, NY, USA). Genomic viral RNA was extracted directly from allantoic fluid or cell culture supernatant with QIAamp Viral RNA Mini Kit (QIAGEN, Valencia, CA, USA). The purified RNA was quantified using NanoDrop (NanoDrop Technologies, Inc., Wilmington, DE, USA). The standard RT-PCR was performed as described previously to amplify the H1_09_, N1_09_, and M_09_ fragments.

### 3.3. Nanomicroarray Assay

Nanomicroarray slides for seasonal H1N1, H3N2, and H1N1/2009 were printed separately, and each capture oligonucleotide was arrayed on CodeLink Activated slides in the triple-spot format (Figure 1A) using the OMNIGrid Accent printer (Genomic Solutions Inc., Marlboroug, MI, USA). Each slide contained 10 identical sub-arrays partitioned by a hybridization gasket, thus enabling 10 tests per slide. Aqueous DNA-modified gold NP-probe and silver staining solutions were purchased from Nanosphere Inc (Northbrook, IL, USA). RNA/DNA samples and 10 nM of the intermediate oligonucleotides were mixed in 100 μL of the hybridization buffer containing 5×SSC, 0.05% sorbitan mono-9-octa decenoate poly(oxy-1,1-ethanediyl), 0.05% Tween-20 and 40% formamide, and applied to the nanomicroarray incubating for 90 min at 40 °C with shaking at 500 rpm. After three washes in wash buffer A (0.5 N NaNO_3_, 0.01% SDS and 0.05% Tween-20) and rinsed once in wash buffer B (0.5 N NaNO_3_), the universal NP-probe was incubated with the slide for 30 min at 40 °C. Then, the slides were stained with the Silver Enhancer A and B solutions for 5 min. The light-scattering signal produced by silver‑enhanced gold NPs was captured by a photosensor and converted to a TIFF image by a Verigene Reader [16]. The resulting TIFF images were analyzed using GenePix Pro 7 software [12,17]. 

## 4. Conclusions

In summary, this study provides evidence that the nanomicroarray assay may be able to differentiate genetic variations among influenza viruses when appropriate samples are available. The assay was able to detect H1N1/2009 and distinguish it from other influenza A viruses. This new method may be useful for simultaneous detection and subtyping of circulating human influenza A viruses and can be rapidly modified to detect other emerging influenza strains in public health settings.
